# Retraction: Maggot extract accelerates skin wound healing of diabetic rats via enhancing STAT3 signaling

**DOI:** 10.1371/journal.pone.0355528

**Published:** 2026-08-10

**Authors:** The PLOS One Editors

After this article [1] was published, concerns were raised regarding results presented in Figs 5 and 6.

Specifically:

- The Fig 5A Bcl-2 N panel appears similar to part of the Fig 6A p-STAT3 M.E. panel
- In Fig 6A, the Cyclin D1 M.E. and Cyclin D1 rhEGF panels appear similar

Corresponding author JL stated that the Fig 5A Bcl-2 N and Fig 6A Cyclin D1 rhEGF panels are incorrect and the Fig 6A p-STAT3 M.E and Cyclin D1 M.E panels are correct. They provided underlying image data for Figs 5A and 6A. Upon editorial review, the *PLOS One* Editors identified additional concerns for these data that call into question the reliability of the reported results.

In light of the above unresolved concerns, the *PLOS One* Editors retract this article.

JL did not agree with the retraction. MLW, ZMY, HCD, HZ, XZ, BY, YY, and PNL either did not respond directly or could not be reached.
